# Extraspectral Imaging for Improving the Perceived Information Presented in Retinal Prosthesis

**DOI:** 10.1155/2018/3493826

**Published:** 2018-04-18

**Authors:** Walid Al-Atabany, Musa Al Yaman, Patrick Degenaar

**Affiliations:** ^1^Department of Biomedical Engineering, Helwan University, Helwan, Egypt; ^2^School of Engineering, Newcastle University, Newcastle upon Tyne NE1 7RU, UK

## Abstract

Retinal prosthesis is steadily improving as a clinical treatment for blindness caused by retinitis pigmentosa. However, despite the continued exciting progress, the level of visual return is still very poor. It is also unlikely that those utilising these devices will stop being legally blind in the near future. Therefore, it is important to develop methods to maximise the transfer of useful information extracted from the visual scene. Such an approach can be achieved by digitally suppressing less important visual features and textures within the scene. The result can be interpreted as a cartoon-like image of the scene. Furthermore, utilising extravisual wavelengths such as infrared can be useful in the decision process to determine the optimal information to present. In this paper, we, therefore, present a processing methodology that utilises information extracted from the infrared spectrum to assist in the preprocessing of the visual image prior to conversion to retinal information. We demonstrate how this allows for enhanced recognition and how it could be implemented for optogenetic forms of retinal prosthesis. The new approach has been quantitatively evaluated on volunteers showing 112% enhancement in recognizing objects over normal approaches.

## 1. Introduction

Humans have six primary senses: touch, taste, smell, hearing, sight, and balance. Of these, vision and touch are arguably the most important. According to the World Health Organization [1], there are thought to be more than 39 million people worldwide who are blind. Visual prosthesis holds promise for a return of functional vision for those conditions which have already caused blindness. For those with photoreceptor disorders such as retinitis pigmentosa (prevalence 1 : 3000), stimulation of the remaining retinal layers is most appropriate. Where the communication circuitry of the eye is no longer functional, as for trauma or glaucoma, neuroprosthetic stimulation would have to be in the visual parts of the brain.

The field of visual prosthesis is perhaps surprisingly old. The first electrical stimulation experiment for the visual cortex dates back to 1929 [2]. In the 1950s, Brindley and Lewin pioneered the first electrical visual cortical prosthesis [3], though progress has subsequently been slow. In 1992, Stone et al. [4] demonstrated that the retinal ganglion (communication) cells were still functional in patients blinded by retinitis pigmentosa. Since then, the biomedical engineering field has invested significant research effort into developing retinal prosthesis. Several research groups and a number of companies around the world are currently engaging in both engineering and clinical efforts.

Traditional electronic approaches have implanted electrodes into the eye or brain to stimulate remaining neurons. In particular, retinal prosthesis has seen primarily subretinal [5–9] and epiretinal [10–12] approaches. The difference relates to the location (resp., underneath or in front of the retina) of implantation of the stimulating electrodes. More recently, there has been an emergence of optogenetic approaches which involves genetically photosensitizing one of the remaining layers of the eye. Such opsins (photosensitization agents) have been successfully expressed in retinal ganglion cells [13], bipolar cells [14], and degenerate photoreceptors [15]. The caveat is a requirement for ultrabright optical stimulation, which has been previously proposed by this team [16, 17]. Nevertheless, key advantages include genetic targeting of retinal subcircuits and that such stimulation could be external to the eye, and not requiring implantation.

Current generations of retinal prostheses return a very basic vision consisting of phosphene percepts [18–21]. Subjects can interpret clusters of such percepts to determine basic visual information such as high-contrast letters and shapes. Optogenetic approaches [17, 22–24] hold much promise, but it may take many years to perfect.

Human visual resolution was first determined by Oesterberg in 1935 to be 120 million rods and 6 million cones [25]. The architecture of the retina then compresses this information to 1 million retinal ganglion (communication output) cells. Such sensor density contrasts with current commercial prostheses which have 1500 stimulators. The retina is spatially structured into different domains—the fovea, macula, and periphery, which have progressively decreasing spatial resolution, but increasing temporal resolution. Typically, electronic retinal prostheses have been placed in a small domain in the macula, but optogenetic variants could potentially project to a broader domain.

Margalit et al. [26] postulated that at least 625 pixels/phosphenes are needed for resolving basic images or the gist thereof. However, beyond spatial resolution, the quality of the image is also determined by the contrast and dynamic range of the stimulators [27]. Finally, the communication protocol is additionally important. The retina has a processing architecture which extracts spatial and temporal derivatives of the scene. Mathematically, a derivative can be positive or negative, but this is difficult for neurons to project. Thus, information is split between on and off pathways whereby information is contained in the differential between the two. If both are stimulated equally, the result will be no net transfer of information. Furthermore, the degenerate retina is very noisy [28]. Finally, if net visual information can be transmitted, the communication pattern of the retinal ganglion cells needs to match the protocol expected by the visual cortex [29].

Clearly, there are many challenges. However, we can take inspiration from invertebrates, which perform remarkably well with only a few thousand light sensors. Although such light sensors have exquisite function [30], the capacity of the subsequent nervous system to process it is low. As such, information content is necessarily low. Nevertheless, their remarkable level of visual function is achieved through contrasting information from dedicated light sensors which extract colour, polarization contrast, and extraspectral ultraviolet wavelengths. As such, bioinspired techniques can be developed which adapt these techniques and use them to improve transmitted images prior to retinal and stimulator encoding [31, 32].

Previously, we have shown that visual recognition of visually impaired subjects can be improved using effective contrast enhancement techniques such as cartoonisation [33]. Furthermore, to deal with the problem of tunnel vision, we developed a nonlinear scene compression approach [34]. In that approach, we compress the most important visual information from a broader visual view into a narrower stimulation view (i.e., tunnel vision). In 2000, Dobelle [35] suggested the use of infrared in his version of optic nerve prosthesis. In that work, it was postulated that infrared could allow the user to switch between views but was not implemented as at that time portable infrared imagers were not available.

**Figure 1:**
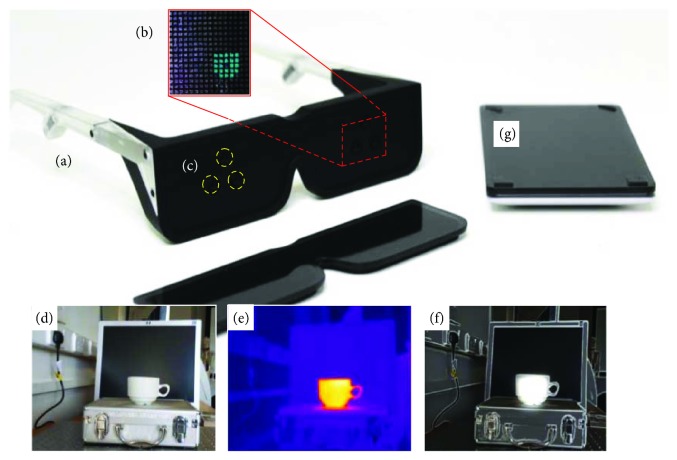
Concept of optogenetic retinal prosthesis, enhanced with extraspectral wavelengths. (a) A concept wearable headset which would project light from (b) a high-density LED array. (c) Cameras which could acquire the infrared, visible, and ultraviolet. (d) Image acquisition form visible, (e) Infrared image, and (f) combined enhanced image prior to retinal processing. (g) A control unit.

In this work, we, therefore, propose to extract thermal information from the scene to assist in the cartoonisation segmentation and contrast enhancement process. This approach would thus integrate both visual and extravisual spectral information within a scene enhancement framework to present the most useful information to the user.  Figure 1 shows the conceptual components of the system.

## 2. Methods

**Figure 2:**
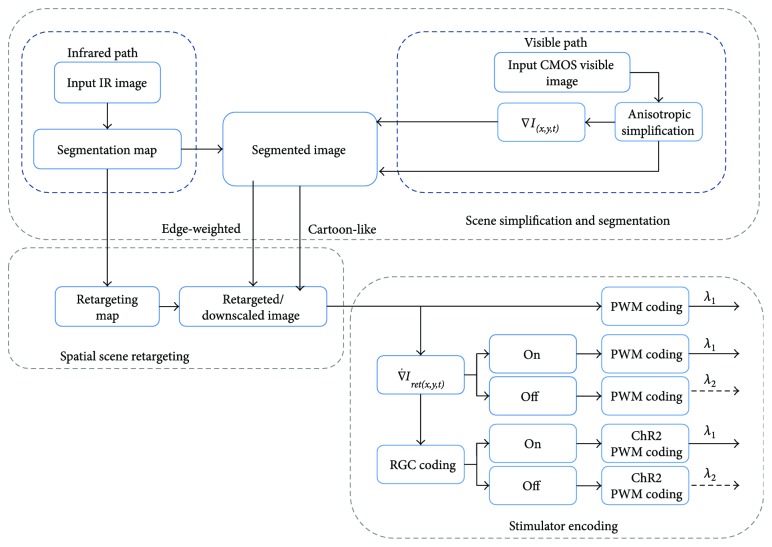
The visible/IR pathways from the two cameras. This includes scene acquisition, contrast enhancement, retargeting, and simulator encoding.

The processing of the visible/IR streams is divided into four main parts according to the flow chart in Figure 2:

1. Image acquisition and preparation—acquisition from both IR and visible cameras and initial gain control and intensity equalisation
2. Boosting the contrast by segmenting the scene—to increase the effective contrast of the scene by reducing irrelevant features (requiring IR enhanced segmentation)
3. Scene retargeting—to fit more information into the visual tunnel by nonlinearly shrinking the scene (requiring IR enhanced segmentation)
4. Stimulator encoding—to provide final required retinal coding and stimulator pulse encoding (not the focus of this paper)

### 2.1. Scene Segmentation and Contrast Enhancement

#### 2.1.1. Generating IR Segmentation Map

The useful range of thermal information is arguably in the range −20 to +100°C. This range can be sensed by blackbody radiation with mid-infrared wavelengths in the 7.8–11.5 *μ*m range, which equates to photon energies of 0.16 to 0.1 eV, respectively. Such energies are below the bandgap of diode semiconductors. As such, microbolometer devices are used to acquire information. Such devices cannot determine spectral differences but infer temperature based on photon flux based on an assumed blackbody radiation profile from the target.

**Figure 3:**
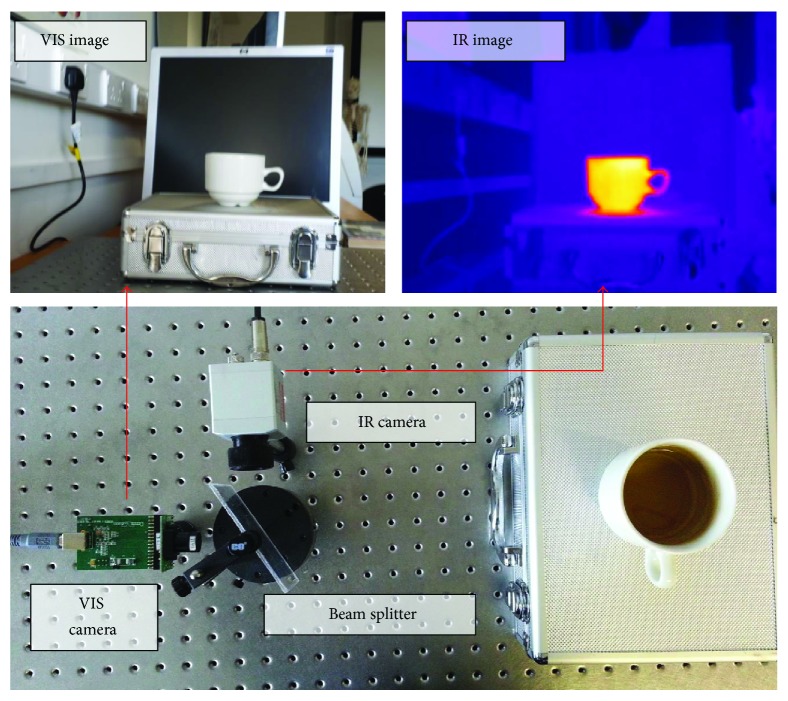
The optical setup of the system. Visible/IR cameras are aligned together through specially designed beamsplitter that reflects the same scene into IR and visible pathways.

We utilised two cameras: infrared (Optris PI 160) and visible (mvBlueFOX-220AC). We optically aligned these perpendicular to each other relative to a beamsplitter which reflected infrared and transmitted visible information.  Figure 3 shows the arrangement of the two cameras and the beamsplitter imaging a hot cup of tea. Hot objects appear to have a greater intensity relative to the background, whereas, for example, a glass of iced tea would have a lower relative intensity. To ensure meaningful information transfer from the IR scene, the automatic gain control needs to be set to a defined range, for example, 0°C to 40°C.

We found that the best approach to separate objects with different temperature gradients was to utilize an exponential scaling function in the infrared intensities. We implemented this on both positive and negative versions of the image. The result is that central temperatures get suppressed in preference to extremes of hot and cold. This is performed as follows:

(1) $$\begin{array}{c} I_{e}=e^{0.025*I},\\ I_{Ne}=e^{0.025*\left(255-I\right)}. \end{array}$$

*I* is the IR image, which is exponentially scaled into *I*_*e*_ and *I*_*Ne*_ is the scaling for the inverse of *I*. Both *I*_*e*_ and *I*_*Ne*_ are combined and exponentially rescaled to further suppress low-intensity pixels. The process separates (segment) the cold/hot objects from the background.

(2) $$\begin{array}{c} {IR}_{map}=e^{0.025*\left(I_{e}+I_{Ne}\right)}. \end{array}$$

**Figure 4:**
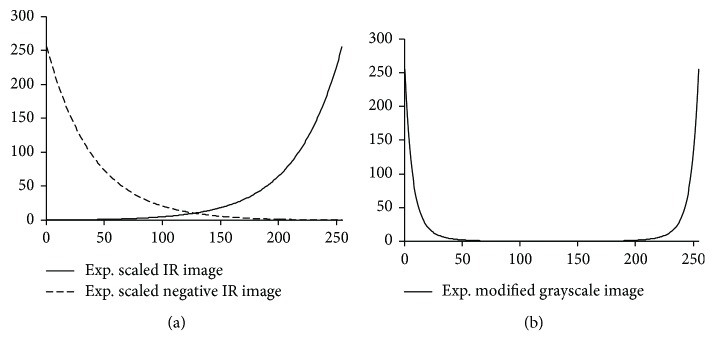
Compressing and stretching the dynamic scale for the IR image and its inverse (a). (b) Exponentially scaling the combined images in (a).

Figure 4 shows the effect of exponentially stretching and compressing an 8-bit grayscale image and its negative. Afterward, we smooth the segmented image to remove any discontinuities using a Gaussian filter.

(3) $$\begin{array}{c} {\hat{IR}}_{map}={IR}_{map}*G\left(x,y\right),\\ G\left(x,y\right)=\frac{1}{2{\pi \sigma }^{2}}e^{-\left(x^{2}+y^{2}\right)/2\sigma^{2}}. \end{array}$$

The segmented image is normalised to be used as a decision map for segmenting and fusing important details from both of the input and enhanced images. Also, it is used in generating an importance map for compressing the segmented image as will be discussed later.

#### 2.1.2. Simplification of the Visual Scene

The primary hypothesis behind scene simplification is that suppression of less important features and textures will accentuate the contrast of more important ones. Subsequent retinal processing will then extract spatial derivatives. As the stimulator array is noisy and with low effective resolution, derivatives of complex scenes [36–40] will look noisy. Thus, simpler, cartoon-like scenes will improve the quality of perception.

Cartoonisation is achieved by first suppressing low-importance textures, then accentuating the edges of key segments. For the first stage, we utilize anisotropic smoothing [41, 42], which smooths textures within higher-contrast boundaries. This is described mathematically as follows:

(4) $$\begin{array}{c} I^{n+1}=I^{n}+\Delta t\left[\nabla \left(C\cdot \nabla I_{H}\right)+\nabla \left(C\cdot \nabla I_{V}\right)\right]. \end{array}$$

*I* is the initial unprocessed image; *c* is the diffusion function, which monotonically decreases as a function of the image gradient value; *∇* represents the gradient operator; *∆t* is the time step (controls the smoothness speed); and *n* is the iteration number. *∇I*_*H*_ and  *∇I*_*V*_ represent the gradients of the image. There are several approaches to calculate the gradients [43]; however, we use horizontal and vertical Sobel operators for simple implementation, from which the gradient *∇* and *C* are calculated as follows:

(5) $$\begin{array}{c} \nabla =\sqrt{{\left(\nabla_{H}I\right)}^{2}+{\left(\nabla_{V}I\right)}^{2}}, \end{array}$$

(6) $$\begin{array}{c} C=\frac{1}{1+\sqrt{{\left(\nabla_{H}I\right)}^{2}+{\left(\nabla_{V}I\right)}^{2}}}. \end{array}$$

Once the anisotropic simplification is complete, we extract spatial gradients of the image:

(7) $$\begin{array}{c} \nabla I=\sqrt{{\left(\nabla_{H}I_{S}\right)}^{2}+{\left(\nabla_{V}I_{S}\right)}^{2}}, \end{array}$$

where *I_S_* is the simplified image from (4)–(6) above. For still images, we simply use the spatial derivatives.

To generate the cartoon-like image, the smoothed grayscale image is then quantized into intensity bins. This clusters regions of similar intensity and also helps compress the dynamic range, which is limited in retinal prosthesis. The quantization relation is given as follows:

(8) $$\begin{array}{c} Q\left(x\right)=q{\left(x\right)}_{nearest}+\frac{\Delta q}{2}\tan h\left(\varphi_{q}\cdot \left(I^{n+1}\left(x\right)-q{\left(x\right)}_{nearest}\right)\right). \end{array}$$

*Q* is the cartoon-like image; Δ*q* is the bin size, the closest bin grayscale to the current pixel *I*^*n*+1^ (*x*); and **φ**_*q*_ is a matrix that controls the sharpness between bins. More description about the algorithm can be found in Winnemöller et al. [37].

To further increase the image contrast, we then combine the negative of the absolute derivative described in (7).

(9) $$\begin{array}{c} I_{cartoon}=\left(1-\left|\nabla I\right|\right)*Q\left(x\right). \end{array}$$

|*∇I*| is the spatial derivative of the anisotropic image normalized to between 0 and 1.

To generate the edge-weighted image, we define a threshold value *K*. Pixels in normalised gradient image with values below this threshold are then normalized to *K*. This value can be modified according to user preference and determines how much of the background features and textures to maintain.

The normalised gradient image becomes a weighting matrix **W** that determines the level of details from the visible image that should be preserved while increasing the brightness of the relevant edges. Then the edge-weighted image can be defined as follows:

(10) $$\begin{array}{c} I_{edge‐weighted}=W*I_{cartoon}. \end{array}$$

#### 2.1.3. Infrared-Assisted Visual Segmentation

The previous IR segmentation map is used here to provide segmentation for the visible image. This is done by creating weighted decision regions from this map by which a linear combination of the pixels in the cartoon/edge-weighted images is used to generate corresponding pixels in the fused image. The generated fused images are as follows:

(11) $$\begin{array}{c} I_{fused\left(edge‐weighted\right)}={\hat{IR}}_{map}*I_{visible}+\left(1-{\hat{IR}}_{map}\right)*I_{visible\left(edge‐weighted\right)},\\ I_{fused\left(cartoon\right)}={\hat{IR}}_{map}*I_{visible\left(cartoon\right)}+\left(1-{\hat{IR}}_{map}\right)*I_{visible}. \end{array}$$

#### 2.1.4. Visual-Assisted Infrared Segmentation

We also explored an optional image modality in which we use the extracted visible gradient information to be fused with the anisotropic diffused infrared image. In this mode, the IR information is simplified using the anisotropic diffusion filter, and a cartoon-like image is generated from it.

### 2.2. Spatial Scene Compression

It is currently difficult for retinal prostheses to present stimulus patterns to the full 140° field of view of the retina. Although it is possible to resize a larger image acquired by a fisheye lens [44], this would make the scene seem further away which makes identifying objects challenging at a lower resolution. Peli et al. also demonstrate scene multiplexing by presenting the edges of a wide field image on top of a narrow field image [45]. However, this approach would present a too-complex image for retinal prosthesis.

We, therefore, want to nonlinearly compress the visual field maintaining the size of the most important features. We, therefore, generate an importance matrix **M** of the image that is used to determine how much each pixel is to be shrunk/compressed. It is composed of two components: the gradient map of the smoothed visible image and the infrared decision map.

(12) $$\begin{array}{c} M=\left|\dot{\nabla }I_{processed}\right|+{\hat{IR}}_{segmented}, \end{array}$$

where $\left|\dot{\nabla }I_{processed}\right|$ is the spatial derivative of the cartoonised or edge-weighted scene. The infrared map gives higher weights for extreme objects' temperatures over the ambient surroundings.

This importance matrix ranks pixel locations for which the shrinking matrix defines how much these pixels in the original image should be shrunk to retarget/compress the image by *K* rows/columns. The shrinkability value of each pixel *S*(*j*) in this matrix is given by

(13) $$\begin{array}{c} S\left(j\right)=\frac{1}{M\left(j\right)*\sum_{j=1}^{M}1/M\left(j\right)}. \end{array}$$

Summing *S*(*j*) over *j* column should equal 1 if *K* is 1. For shrinking the image by *K* columns or rows, we simply rescale the map to the desired shrinkage range.

The generated shrinkability map is used after that to retarget either the cartoon or the edge-weighted visible images to the desired size using Fant's algorithm [46]. It maps a limited 2-D matrix of discrete input pixel to another limited matrix. The full description of the scene retargeting approach can be found in our previous paper [34]. According to the number of array stimulating points, further linear image rescaling can be used to scale the nonlinearly retargeted scene into smaller sizes.

### 2.3. Retinal and Pulse Coding

In our previous work [47], we described three different scenarios for retinal prosthesis: stimulating the reinnervated cone cells in the macula, stimulating bipolar cells, or stimulating retinal ganglion cells. Figure 2 shows the process flow which can be summarised as follows:

- Cone stimulation: this would utilize either the edge-weighted or cartoon-like image, which should be controlled according to the user preference, followed by LED pulse coding.
- Bipolar stimulation: here we could use the derivative of the ones used with the cone stimulation. The result could be split into on/off pathways, followed by LED pulse coding.
- RGC stimulation: this would be similar to bipolar stimulation, except with the further possibility of driving the spike code using an Izhikevich neuron model.

Once an event occurred, we need to generate a pulse to stimulate the ChR2 encoded cells. The pulse width depends on the intensity of the stimulus and the sensitivity of the ChR2 encoded cells. As channelrhodopsin has dark and light adapted states, using variable pulse width is more efficient than using fixed pulse [48]. Once stimulated, ChR2 goes into its less efficient light-adapted form requiring around 50 ms to recover. We, therefore, determine two pulse widths: short (5 ms), in the case of no action potential stimulus in the previous 50 ms and long (10 ms), in the case of a previous action potential stimulus in the previous 50 ms.

## 3. Results and Discussion

**Figure 5:**
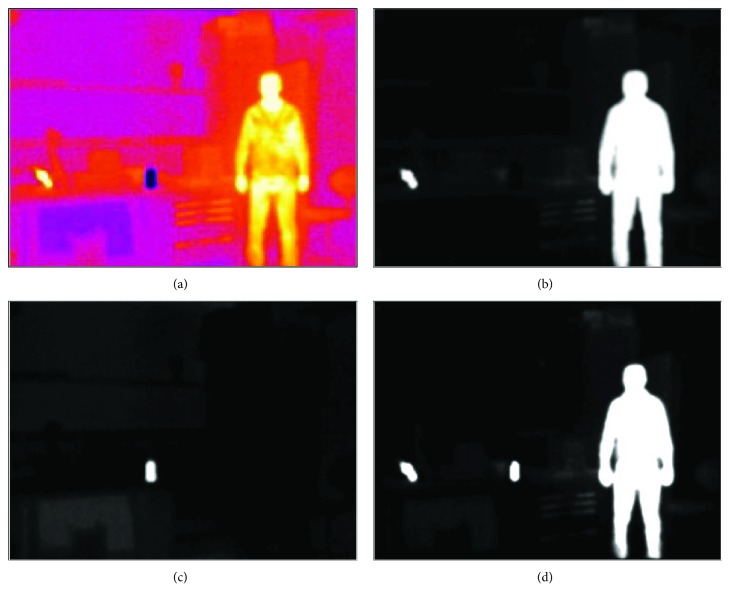
Pathway for the infrared image. (a) Captured IR image. (b, c) Segmented hot and cold objects. (d) Segmentation map.

Figure 5 shows an infrared scene for a standing person whose temperature is higher than the ambient. Objects with temperatures higher or lower than the surroundings are extracted using the exponential scaling approach described in the methodology section. This boosts brighter objects to higher grayscale values while suppressing darker objects. The segmenting map image is generated by combining the cold and hot images as shown in (d).

**Figure 6:**
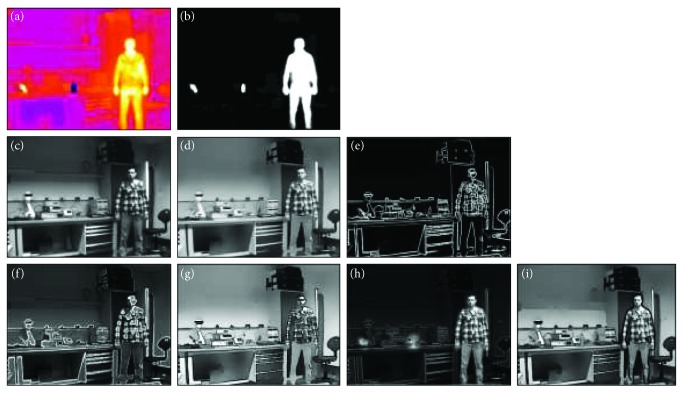
The output of different stages of the flow chart shown in Figure 2. IR path: IR image (a) and its segmentation map (b). Visible path: visible (c), anisotropic diffusion (d), and gradient images (e). Enhanced: the edge-weighted (f), cartoon-like (g), segmented edge-weighted (h), and cartoon-like enhanced images (i).

Figure 6 shows the output of each stage of the scene processing platform shown in Figure 2. The infrared image and its derived segmentation map are shown in the top row. The original image, its anisotropic smoothed version, and the extracted gradient image are shown in the middle row. The scene simplification stage is very effective in enhancing key features for prosthesis systems with low spatial resolution. Figure 6(f) shows the two enhancement techniques we use in this paper, cartoon-like and edge-weighted images. Controlling the level of details in the edge-weighted image can be done by varying the value of *K*. Figure 6(i) shows the segmented edge-weighted and cartoon images which were generated from the infrared segmentation map.

**Figure 7:**
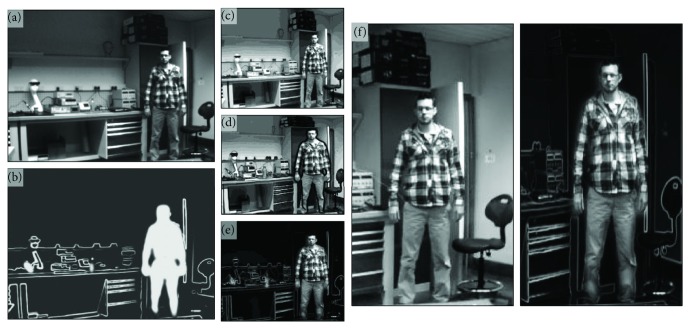
The effect of segmentation process on image retargeting. The left column shows the original scene (a) and the importance map for the retargeting process (b). The middle column shows the linearly scaled image (c), the nonlinear retargeted image of the segmented cartoon (d), and edge-weighted images (e). (f) shows a close-up of the individual demonstrating the effect of nonlinear retargeting on the size of important features.

**Figure 8:**
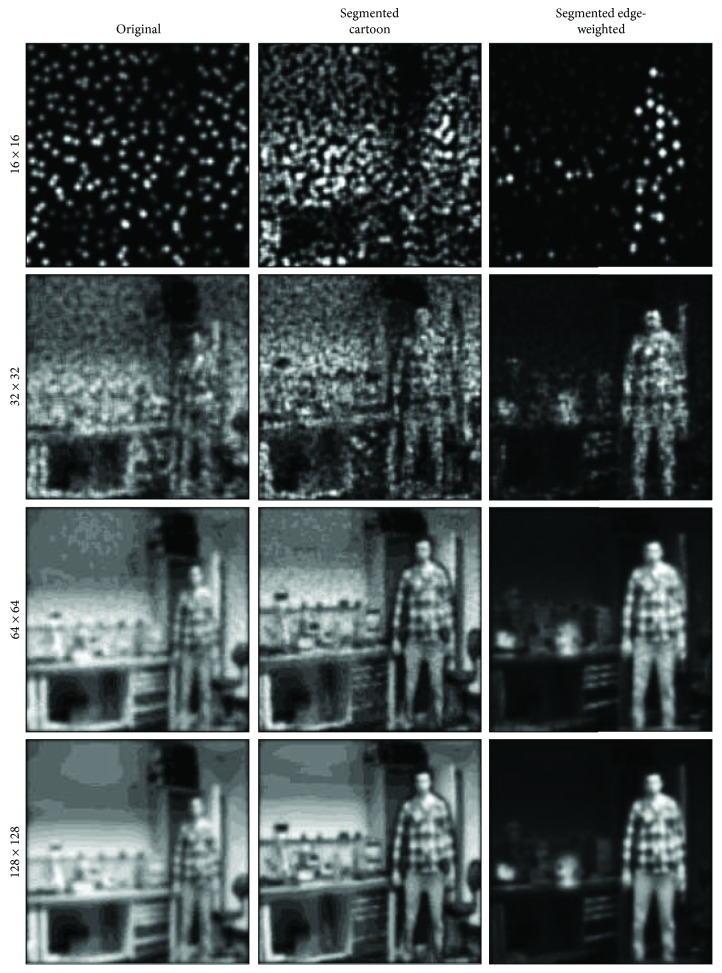
Simulating the vision at different sizes of stimulating arrays. A simulation for what a subject with different stimulating retinal prosthesis arrays (16 × 16, 32 × 32, 64 × 64 and 128 × 128) would perceive is shown from the top row to the bottom row. The left column is a simulation for the original scene and the middle and right columns are for the segmented cartoon and edge-weighted images, respectively, after nonlinearly retargeted by 30% in both directions.

Figure 7 shows the effect of our nonlinear retargeting algorithm after the segmentation process. The effect of nonlinear retargeting approach compared to the linear scaling process is clear from the middle column that shows that the dimensions of relevant objects are kept intact while those of the irrelevant objects are not.  Figure 8 shows the efficiency of our scene optimisation and simplification approach by simulating the vision when different sizes of stimulator arrays are used. We can see that when using the original image with low stimulating array size (e.g., 32 × 32 and even 16 × 16), objects of the foreground and background are fused together. This is not the case when using the segmented edge-weighted image which maximizes the information perceived from important objects.

Our hypothesis is that at lower resolutions, the most useful function is enhanced mobility, that is, having an awareness of objects relative to the user. This can be achieved using the edge-weighted approach which increases the contrast of objects by highlighting the edges of important features while suppressing irrelevant pixels in the scene. Going to higher effective resolutions allows more information to be perceived as shown from the third and fourth rows. At this point, there is perhaps a crossover to cartoonisation being more useful as it presents more of the background features. In the end, we envisage this to be up to the patient's individual preference.

In addition, our scene optimization algorithm was objectively evaluated in recognizing certain actions in videos using real subjects. We have tested the algorithm on 15 volunteers. The participants were each asked to watch 5 video files (ranging from 21 to 27 sec) at two scales of resolutions (16 × 16 and 32 × 32) with and without our scene-enhancing algorithm, resulting in an overall viewing of 20 video files. Video files are displayed randomly to the participants. The participants were asked to recognize actions and count the number of detected persons in the video files.  Table 1 shows the number of persons and actions' description of each video file. The percentage of detected persons and recognized actions was measured with respect to the total number of persons and actions (a total of 13 persons and 11 actions occurred in the 5 video files).

**Figure 9:**
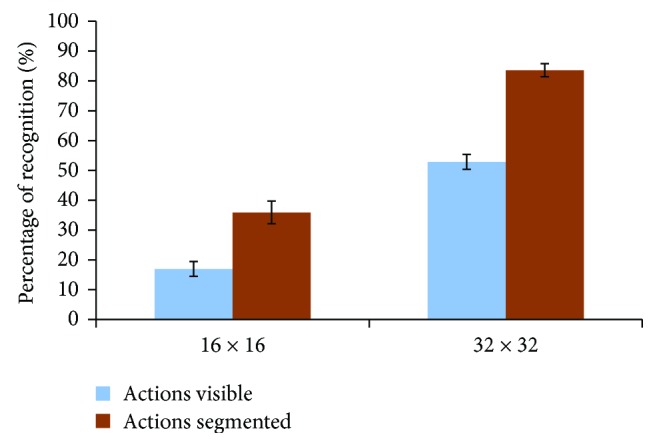
The efficiency of the optimization algorithm in recognizing actions in dynamic videos. Simulated video perception for different stimulating array sizes (16 × 16 and 32 × 32) have been presented to the candidates and they asked to recognize the objects. Greater improvement in number of recognized objects has been achieved using our scene-optimization algorithm. The error bars represent the standard error of the data.

The percentage of recognized actions in the presented videos for the candidates with respect to the actual number of actions is shown in Figure 9 with and without using our scene-optimization approach. The percentages are calculated for a simulation of the perception of two sizes of stimulating arrays, 16 × 16 and 32 × 32. We can see that there is a significant difference (*P* = 0.002, one tail *t*-test) between the recognized actions/objects in the original videos and those optimized using our proposed approach. This gives an enhancement of object recognition by more than 112% for the stimulating array size of 16 × 16. The enhancement percentage has been calculated based on 14. Additionally, using larger sizes of stimulating array 32 × 32, the recognition rate significantly increased (*P* = 3.4596*e*^−08^) by 58%.

(14) $$\begin{array}{c} \%\text{ of }enhancement=100\times \left(\frac{N_{en}}{N_{ac}}-1\right). \end{array}$$

*N*_en_ is the number of recognized actions using our scene-enhanced approach. While *N*_ac_ is the number of recognized actions from the actual/unprocessed scene.

**Figure 10:**
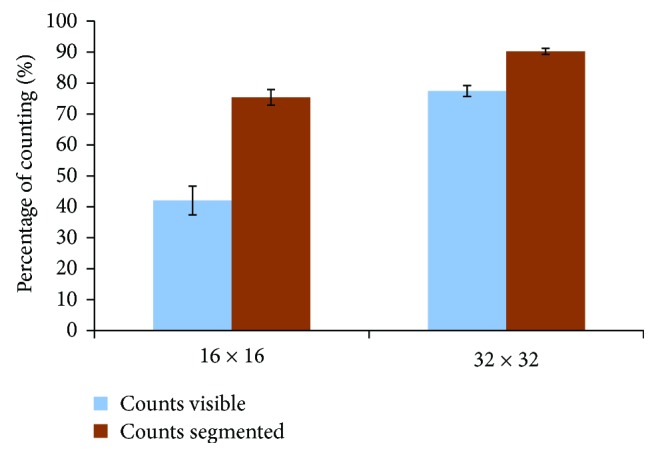
The efficiency of the optimization algorithm in identifying and counting persons in dynamic videos. Simulated video perception for different stimulating array sizes (16 × 16 and 32 × 32) have been presented to the candidates and asked to count the number of persons in each video. Greater improvement has been achieved using our scene optimization algorithm. The error bars represent the standard error of the data.

We can get the same observation from Figure 10 which shows the percentage of correctly identified persons in the presented videos for the candidates with respect to the actual number of persons. We can see that there is a significant enhancement (*P* = 1.9706*e*^−05^) in the counting number using our scene enhancement approach when simulating the 16 × 16 stimulating array. Moreover, the enhancement significantly (*P* = 1.7667*e*^−05^) exists when increasing the stimulating array size to 32 × 32.

**Figure 11:**
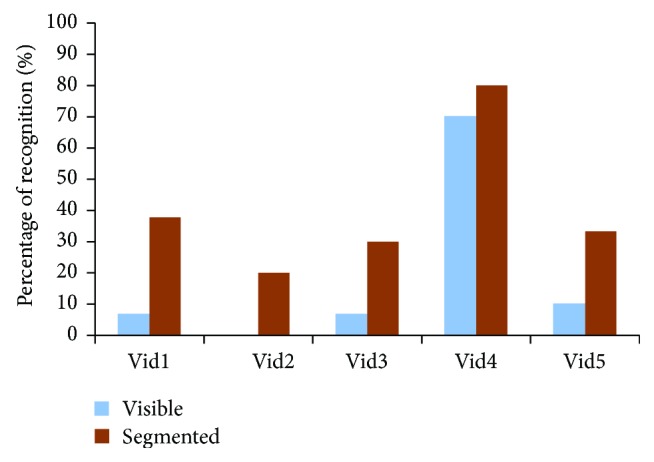
The efficiency of the scene optimization algorithm in recognizing actions for each video when simulating the perception of using stimulating array size of 16 × 16.

**Figure 12:**
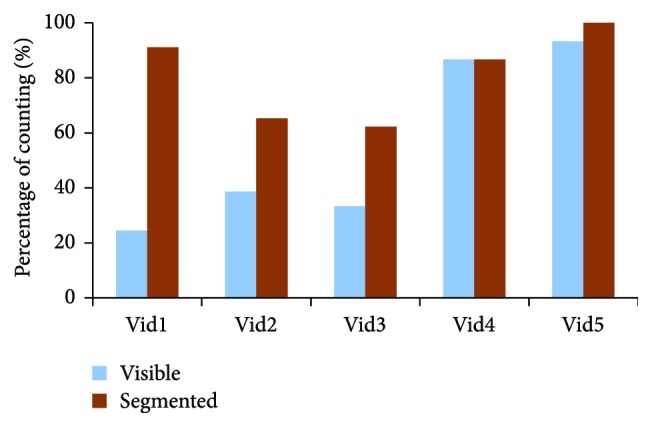
The efficiency of the scene optimization algorithm in identifying and counting subjects for each video when simulating the perception of using a stimulating array size of 16 × 16.

Candidates found that busy sequences such as the second one were very difficult to recognize and their actions difficult to describe due to limited resolution as shown from Figure 11 and Figure 12. The continuous movement of the five subjects in this video made it very difficult for recognizing and differentiating between persons and the actions they were doing, while this was not the case in simple sequences like the fourth video as it was recognizable in both versions of resolutions, 16 × 16 and 32 × 32.

**Figure 13:**
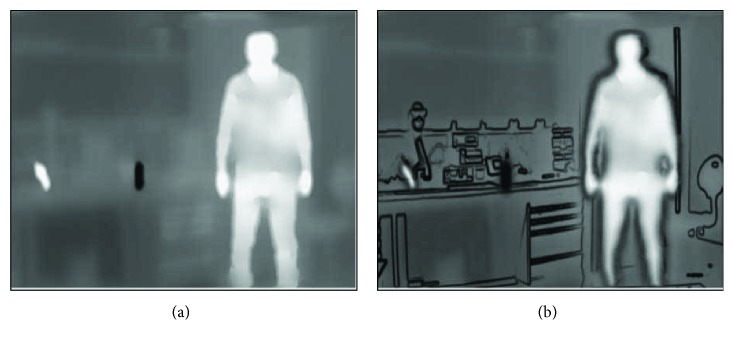
Visually enhanced IR segmentation. The base IR image (Figure 5(a)) above is smoothed with the anisotropic method (a) and then cartoonised via edge overlay from the visual scene (b).

In the case of visible-assisted segmentation of IR, the nature of the information is different to the visible. The absolute intensity becomes the key important feature. As such, we have used anisotropic diffusion to smooth the scene, as can be seen in Figure 13(a). We then utilize the segmented edges from the visible to better outline those features as can be seen in Figure 13(b). This allows the user to see key hot/cold objects.

**Figure 14:**
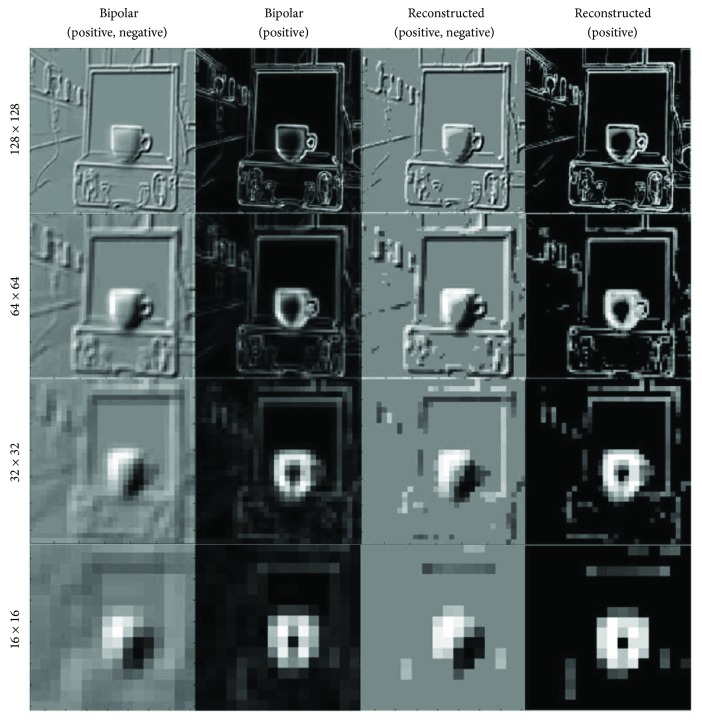
Bipolar cell output and reconstructed image from RGC. A simulation for what a subject with different stimulating retinal prosthesis arrays (128 × 128, 64 × 64, 32 × 32, and 16 × 16) would perceive the full bipolar image and the approximate (positive values only), columns one and two. Columns three and four show the reconstructed images from the RGC for full bipolar and approximate bipolar.

Figure 14 shows simulation for the output of bipolar cells and a reconstructed scene from RGC. We show the results at different resolution starting from 128 × 128 down to 16 × 16. The output of bipolar cells is shown in two columns at different sizes; the first column shows the exact output when using the on and off bipolar components of the image. However, the second column shows the absolute gradients of the on/off image for situations where only one pathway is to be stimulated. Columns three and four show the reconstructed images from both the on/off and on-only RGC using the time to first spike encoding approach.

Results from our proposed system showed the importance of using dual spectrum imaging system in segmenting, simplifying, and retargeting the scene before spike coding. We also demonstrated the effect of increasing stimulator resolution on scaling the image sent to the patient (assuming retinal coding).

Ultimately, this work has shown efficacy in scenes whereby the camera is static and objects in the scene move. In this situation, creating an importance map is straightforward. However, in the situation where the camera moves relative to the environment, the movements of all features would need to be subtracted from the background movement. This background movement would then have to be calculated from the optic flow analysis on the images or from accelerometer motion sensors.

The key to implementability of any image processing front-end system is its function on portable processing systems at full video frame rate. We used the Matlab platform to build our scene optimization algorithm. The processing was implemented on a laptop computer, with a 2.5 GHz Intel i7 processor. We did not use any GPU-assisted processes, and thus, all the operations are processed through the CPU/FPU. Although, we have achieved a frame rate of 27 frames/sec.

Also we have deployed the algorithm in Raspberry Pi for direct comparison of the overall processing time. Direct deployment achieved 11 frames/sec for a frame size of 64 × 64. However, as is generally accepted in the graphics processing community, parallel processing using GPU architectures can speed up the processing time in the range of 10–100x depending on the level of parallelism of the program. However, this is beyond the scope of this paper.

We explored the direct fusion of IR, UV, and visible information. A concept video can be found here [49]. However, while we found that individually such wavelengths can be interesting, in most cases, it qualitatively tended to degrade rather than enhance the usefulness of the image. As such, we feel that the best approach is to use IR to support segmentation of visible. With regard to UV imaging, it is certainly interesting to see flowers in the ultraviolet spectrum. But it has little function indoors (low UV light levels), and as human beings, we are not primarily interested in pollen collection. As such, we could not find a way to justifiably integrate it into a retinal prosthesis imaging system.

The Optris camera we used in this work has a power consumption of between 0.5 and 1 W. More recently has been the advent of portable mobile phone-based microbolometer imaging systems with power consumption as low as 150 mW [50]. We would expect this to improve further in the coming years. We estimate the total power for our prosthetic system to be similar to that of smartphones or tablets, that is., ~1 W. This would be feasible to recharge on a daily basis utilizing a 30 WHr battery (3x that of mobile phone batteries).

## 4. Conclusions

In this paper, we present a multispectral imaging interface for a visual prosthesis. We have demonstrated a method which is not simply separate spectra or an image fusion. Rather, we demonstrate how the visual image can be used to segment and cartoonise the infrared scene and how the infrared can be used to segment, cartoonise, and compress the visible. We believe that our method usefully combines information from infrared and visible to best convey the most useful information to someone with poor vision. It may in the future also prove useful when integrated into assisted vision devices for the visually impaired (but not blind).

## Figures and Tables

**Table 1:** Description of the subjects and actions of each video.

Video number	Number of persons	Actions
1	3	A person waving his two hands A person changing his standing position to sitting A moving person from left to right of the frame

2	5	A person holding a jacket from a table A person wearing a jumper

3	3	Two persons standing and talking to each other A subject holds a cup from a table and drinks A person raising his hands

4	1	A waving hand while opening and closing it

5	1	A person holding a bottle while drinking A person waving his two hands
